# Everybody's Page

**Published:** 1889-07-20

**Authors:** 


### Everybody's Page.

The salt works of Spain, in the Balearic Isles, the Bay of Cadiz, and elsewhere, turn out about 300,000 tons of salt yearly distilled from sea-water.

The Chinese have a superstitious idea that it is a solemn duty to keep the body intact, and for this reason they have a strong objection to anything in the nature of a surgical operation. If they are persuaded to submit to the amputation of a limb they invariably keep the severed limb, which in due time is buried with its owner. Some of the more barbarous among them eat it, on the principle that what is taken from the body should be returned to it.

The cork oak grows well in California, and though it is a crop that has to be long waited for, it is a remunerative one when it comes. One tree will sometimes furnish as much as half a ton of cork, and as a pound of cork is sufficient for twelve dozen champagne corks, it is easy to calculate how profitable the yield is. It takes ten years before the cork bark is thick enough to be of use, but, fortunately, the tree does not die when its outer garment is taken off; it merely sets to work to grow another coat of bark for the destroyer.

The following remedy for hydrophobia is given by Mr. G. Pigot, as having long been used successfully in the Island of Skye: Boil one handful of the leaves of datura stramonium, or thornapple, in one pint of water, till the mass is reduced to one-half. Strain the fluid through a fine white cloth, and administer it in one dose; if difficult to swallow, it must be forced down the throat. Before long violent paroxysm will ensue, accompanied by copious perspiration; then sleep for eight hours will follow, and the patient will be free from rabies.

Inhalation of chloroform is the latest cure suggested for phthisis. It has been tried successfully by Dr. Mansfelde, an American physician, whose communication on the subject to the *Medical Record* shows parenthetically how far the co-operation of man and woman has gone in the United States. Having thought out the theory of the inhalation to its conclusion, Dr. Mansfelde says: I at once informed my wife, who administers chloroform for me at all times, from the operation of ovariotomy to the lancing of a felon." Dr. and Mrs. Mansfelde ought to be a happy pair.

The superstition of "thieves' candles" is a very widespread and horrible one. It consists in the idea that if candles be made from human fat, those who use them are rendered invisible. A case came before a Russian tribunal recently, in which four men were easily caught when engaged in a robbery because they believed in the efficacy of the thieves' candles they were using and kept no watch. At the trial it came out that they had committed a murder some time before the robbery in order to obtain the material for the candles.

"Sweet are the uses of advertisement!" So our hot and thirsty dogs should exclaim this summer weather, and invoke a canine blessing on the proprietor of Hudson's soap, who is placing small troughs all over London for their benefit. The troughs bear the inscription, "Drink, puppy, drink," along with a casual allusion to the merits of Hudson's soap. We should suggest to our canine friends that as a drink in time may save them from the horrid imputation of madness, and a visit to the lethal chamber at Battersea, they ought, as a matter of gratitude, to do their best to introduce Hudson's soap to the families they belong to, by insisting that no other shall be used for their own bath.

Several cases of poisoning have recently been caused by the consumption of chocolate and other confectionery that had been wrapped in what is called tin-foil, but really contains a large percentage of lead.

The bristles used in the manufacture of brushes come mostly from Russia. In this St. Petersburg is the largest market in the world, though Poland, Germany, Belgium, France, and Chicago also supply large quantities, which, however, are of inferior quality.

The disease in wood called dry rot is contagious. It may spread from diseased wood to sound, and it has been surmised that the disease may be conveyed by tools that have been employed on diseased wood. We shall soon have antiseptic woodcutting among the refinements of modern life.

What are our horses coming to! Recently a horse was seen at the door of a public-house drinking with evident enjoyment the beer in a pewter mug, which his master held to his mouth. If anything likes this becomes customary it will be necessary for some high principled member of the equine race to start a "horses' total abstinence society," and in choosing a cab at night we shall look carefully to be sure that the horse as well as the driver is sober.

"And remember," said the doctor to the young man, after giving him a prescription, "if you wish to get well, take only one cigar after each meal." "Very well," said the patient sadly, and went away. A few weeks later he returned to say that his health was greatly improved. "But I wish, doctor," he added, "that you could let me off that cigar after each meal. You see I had never smoked till you told me to, and I don't really think I am any the better for it.

The method in which the most delicate perfumes are obtained from flowers is not of the most æsthetic nature. The flower petals are spread over glasses which have previously been covered with a quarter-inch layer of fat. The glasses are then shut tightly into wooden frames, and before long the fat absorbs all the perfume. The next process is to cut up the fragrant fat into small pieces and put these in alcohol. The perfume at once deserts its oily protector and unites with the alcohol. It is then fit for the market.

It is possible that undue blame is sometimes cast on parents and guardians in the case of child-suicides. It is not inevitably the case that great injustice and cruelty are required to drive a child to self-destruction. Quite as often a morbid habit of mind, a constitutional taint, together with the lack of proportion in his estimate of things and their consequences, make a sensitive child commit suicide for some trifling fault or accident which another would take lightly, and which, perhaps, the much-blamed parents would not have punished at all.

A recent experience of Dr. Iterson, a German physician, shows the danger of performing operations that require anæsthesia by gas light. It appears that the fumes of chloroform, mingling with those of the gas, form a compound that tends to produce asphyxia in those within its power. He records one death, which he regards as due to the combination of chloroform vapour with the products freed by the combustion of gas. In another case the experience was very peculiar; while under the influence of the anæsthetic the patient's respiration was unaffected. It was only after he regained consciousness that the symptoms of asphyxia set in, and free ventilation of the room promptly removed them.

